# Phenotypic characteristics of familial glucocorticoid deficiency (FGD) type 1 and 2

**DOI:** 10.1111/j.1365-2265.2009.03663.x

**Published:** 2010-05

**Authors:** Teng-Teng L L Chung, Li F Chan, Louise A Metherell, Adrian J L Clark

**Affiliations:** Centre for Endocrinology, Barts and the London School of Medicine and Dentistry, Queen Mary University of LondonLondon, UK

## Abstract

**Context:**

Familial glucocorticoid deficiency (FGD) is a rare autosomal recessive disorder as a result of mutation in genes encoding either the ACTH receptor [melanocortin 2 receptor (MC2R)] or its accessory protein [melanocortin 2 receptor accessory protein (MRAP)[. The disorder is known as FGD type 1 and 2, respectively.

**Objective:**

The aim of the study was to compare the phenotype/genotype relationships between FGD 1 and 2.

**Design and patients:**

Forty patients with missense MC2R mutations and 22 patients with MRAP mutations were included. Forty-four of these patients had been referred for genetic screening and 18 were patients published by other authors.

**Results:**

The median age at presentation for FGD type 1 was variable at 2·0 years; range 0·02–16 years, and this was associated with unusually tall stature, mean height SDS + 1·75 ± 1·53 (mean ± SD). In contrast, FGD type 2 presented at a much earlier median age (0·08 years; range at birth to 1·6 years) (*P* < 0·01) and patients were of normal height SDS + 0·12 ± 1·35 (*P* < 0·001). No differences in baseline cortisol or ACTH levels were seen between FGD types 1 and 2.

**Conclusion:**

FGD type 2 appears to present earlier. This may reflect the functional significance of the underlying mutations in that all MRAP mutations are nonsense or splice site mutations that result in abolition of a functional protein, whereas most of the MC2R mutations are missense mutations and give rise to proteins with some residual function. Tall stature is associated with mutations in MC2R but not in MRAP. There were no other significant clinical distinctions between the two.

## Introduction

Familial glucocorticoid deficiency (FGD) or hereditary unresponsiveness to ACTH is characterized by isolated glucocorticoid deficiency. It is an autosomal recessive disorder resulting from ACTH resistance, typically presenting between the neonatal period and late childhood; with hyperpigmentation, hypoglycaemia and seizure.^1^–^4^ The first inactivating melanocortin 2 receptor (MC2R) mutations in FGD were described in 1993.^5^,^6^ Since then, multiple mutations have been identified throughout the receptor, the majority of which are homozygous or compound heterozygous missense mutations; FGD resulting from MC2R mutations accounts for ∼25% of all FGD and is now known as FGD type 1 (OMIM#202200). FGD type 2 (OMIM*609196) describes a group of patients with normal MC2R but with mutation in the melanocortin 2 receptor accessory protein (MRAP) which is required for MC2R trafficking and function^7^ and this accounts for ∼20% of FGD and is now known as FGD type 2.^8^

There are at least 25 missense mutations of MC2R identified in FGD type 1. *In vitro* functional studies have shown that the majority of MC2R mutations do not function because they fail to traffic to the cell surface.^9^ Nine mutations of MRAP in FGD type 2 have been reported to date, all of which are splice site or nonsense mutations and are predicted to produce proteins lacking the transmembrane domain essential for interaction with MC2R.^7^,^9^–^11^

This paper compares the phenotypes of all previously reported (and some unreported) FGD type 1 and 2 patients and their biochemical findings. As MRAP is expressed more widely than MC2R,^7^ and because of evidence that it may interact with other melanocortin receptors,^12^ we have considered whether the FGD type 2 phenotype may differ from that of FGD type 1, perhaps reflecting a broader spectrum of activity than that of the MC2R. Of particular relevance is the report that a patient with FGD type 2 was significantly obese from childhood, which prompted the authors to speculate that MRAP may have a role in the appetite regulatory function of MC4R in the hypothalamus.^11^

## Patients and methods

### Patients

One hundred and sixty-four patients were referred to us from 1993 to 2008 for genetic screening with a clinical diagnosis of FGD characterized by elevated plasma ACTH, and low or undetectable cortisol in the absence of overt mineralocorticoid deficiency. Other causes of adrenal insufficiency were excluded. From this cohort, 26 patients with FGD type 1 and 18 with FGD type 2 were identified. All clinical details and information given by the referring physician were included in the analysis.

We also performed a PubMed search for all genetically identified FGD type 1 and 2 cases reported until December 2008 and this resulted in a further 18 patients being included in this study.

### Mutational analysis of MC2R and MRAP

Genomic DNA was extracted from peripheral leucocytes from the index patients. Polymerase chain reaction (PCR) amplification was performed using specific intronic primers designed to MRAP and MC2R genes (primer sequences and PCR conditions available on request). All mutations were confirmed with a second PCR and parental DNA where possible. PCR products were sequenced using an ABI 3700 genetic analyser, according to the manufacturer’s protocol (Applied Biosystems, Foster City, CA, USA).

### Statistical evaluation

Data were tested for normality on spss software Version 16 (SPSS Inc., Chicago, IL, USA). For normally distributed data, the two groups were compared using independent *t*-test. Values are stated as the mean ± sd, unless otherwise stated. Significance was defined as *P* < 0·05.

## Results

**Fig. 1:**
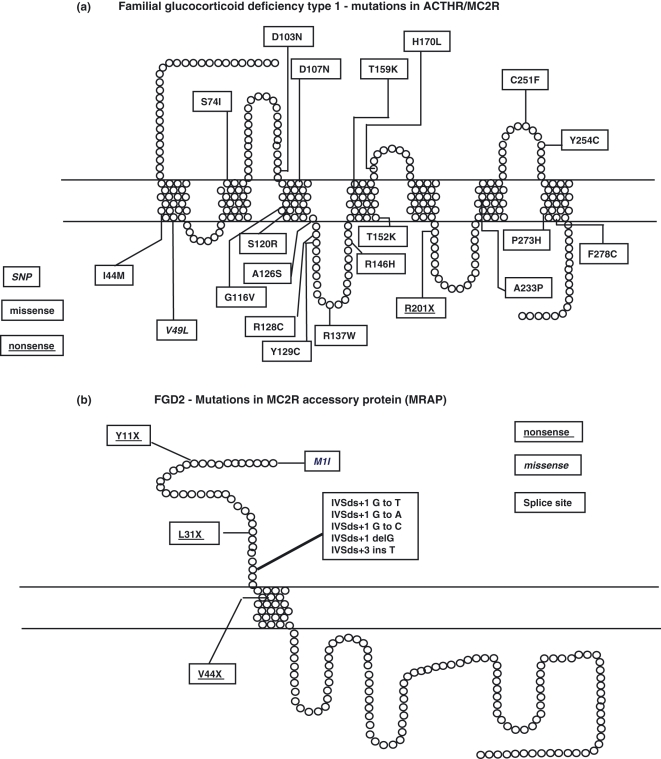
Pseudostructural plot of MC2R (a) and MRAP (b) showing the positions of the mutations included in this analysis. Copyright permission obtained from Elsevier publishing group.^36^

We included in the analysis 40 FGD type 1 patients with 21 different MC2R mutations in homozygous or compound heterozygous form and 22 FGD type 2 patients with 9 different MRAP mutations (Fig. 1). The majority of the MC2R mutations have been previously reported,^13^–^25^ and of these the S74I mutation was present in 18 patients. All nine mutations have previously been reported for FGD type 2.^7^,^11^,^26^,^27^

Data included in the comparisons were the age of presentation, height (in SDS), weight (in SDS) of subjects at presentation, biochemical parameters including ACTH and cortisol levels and response to short synacthen testing (where available). Insufficient data were available to comment on bone age, final adult height or adrenal androgen production.

### Age at presentation

**Fig. 2:**
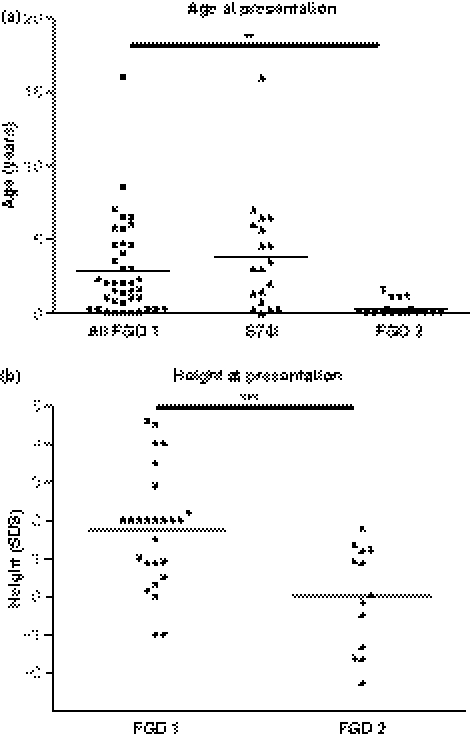
Age and height of FGD patients at presentation. (a) Age (years) of all patients with FGD type 1, those patients with the S74I mutation, and FGD type 2 are shown. The horizontal line represents the mean. (b) Height (in SDS) of all patients with FGD type 1 and FGD type 2 are shown. The horizontal line represents the mean. **P < 0·01; ***P < 0·001.

FGD type 1 presents with a variable age of onset, median age is 2·0 years (range 0–16 years; mean age 3·11 ± 3·40 years), while FGD type 2 presents at an earlier age with median onset at 0·08 years (range 0–1·6 years; mean age 0·31 ± 0·51 years), *P* < 0·01 (Fig. 2a). The most common MC2R mutation, homozygosity for S74I, appears to display a wide spectrum in age of presentation, with the median age of onset 3·2 years (range 0–16) which does not differ from that of other patients with FGD type 1 (Fig. 2a).

### Height and weight at presentation

**Fig. 3:**
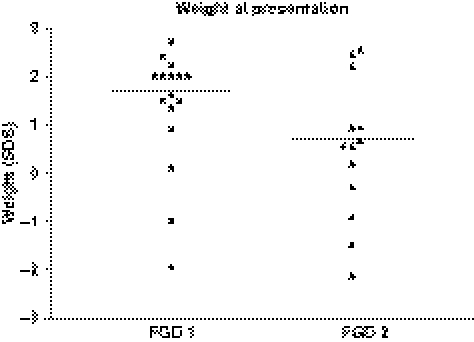
Weight of FGD patients at presentation. The weight (in SDS) of all patients with FGD type 1 and 2 are shown. The horizontal line presents the mean. There was no statistical significance detected.

The height of patients with FGD type 1 has previously been noted to be unusually tall.^2^,^16^,^28^ In FGD type 1, height SDS at presentation was +1·76 ± 1·52 (mean ± SD) and in FGD type 2 height SDS at presentation was +0·12 ± 1·35, *P* < 0·001 (Fig. 2b). Not all weight measurements were available for FGD 1. Where data were present, the mean weight SDS for FGD type 1 (*n* = 19) at presentation was +1·7 ± 1·46 and in FGD type 2 (*n* = 14), the mean weight SDS at presentation was +0·718 ± 1·62 (NS) (Fig. 3).

### ACTH/cortisol at presentation

**Fig. 4:**
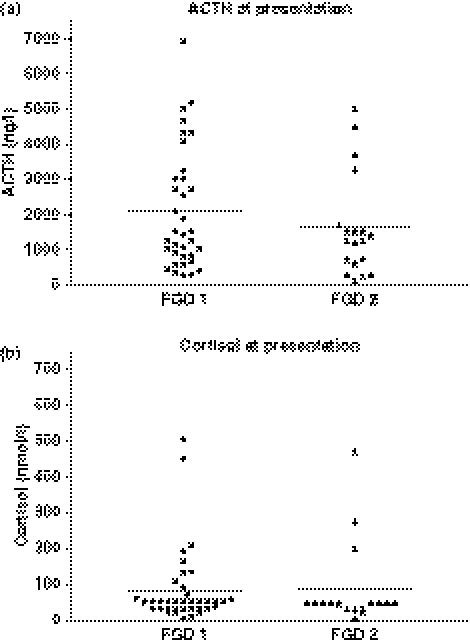
Plasma ACTH and cortisol at presentation. (a) ACTH (ng/l) and (b) cortisol (nmol/l) for all patients with FGD type 1 and 2 are shown. The horizontal line represents the mean. No statistical significance was detected in the two groups.

The median plasma ACTH (range) at presentation for FGD type 1 was 1409 (250–6888) ng/l and 1250 (108–4500) ng/l for FGD type 2 (NS) (Fig. 4a). Similarly, the basal cortisol at presentation is shown in Fig. 4b, with FGD type 1 median cortisol 50 (8–502) nmol/l and FGD type 2 median cortisol 50 (5·5–200) nmol/l (NS).

## Discussion

The diagnosis of FGD is based on clinical findings and patients usually present with hypoglycaemia, seizure, jaundice, hyperpigmentation, failure to thrive and frequent or severe infections. The biochemical findings are a markedly elevated plasma ACTH in the presence of low cortisol but with a preserved mineralocorticoid production and are characteristic of ACTH insensitivity. There are two genetically identified causes of FGD – mutations in the MC2R and mutations in MRAP known as FGD type 1 and 2, respectively. This is the first phenotype–genotype comparison between FGD types 1 and 2.

We have shown in *in vitro* studies that missense mutations in the MC2R have varying degrees of impaired trafficking from the endoplasmic reticulum (ER) to the cell surface resulting in reduced receptor expression (20–100% when compared with wild-type) and ACTH signalling.^9^ Consequently, there is often some protein with residual function. In contrast, MRAP is required in the earliest stages of MC2R processing and in the absence of MRAP protein, the receptor is retained within the ER.^7^,^9^,^29^ G protein-coupled receptors which are trapped in the ER are subject to degradation by retrotranslocation and proteasomal degradation.^30^ All MRAP mutations reported so far will prevent translation of the full-length protein. There is evidence that MRAP may also play a role in ACTH binding and/or signalling,^31^ and without MRAP, the MC2 receptor will not traffic efficiently to the cell surface leading to complete failure of receptor function. Studies of the expression of the MRAP gene in humans reveal a wider pattern of expression than that of the MC2R.^7^ This suggests that MRAP may have additional roles to those related to the MC2R, and we have demonstrated an interaction with each of the melanocortin receptors.^12^ Therefore, examination of any discrepancies between FGD types 1 and 2 may provide important clues to other functions of MRAP. It has been proposed that MRAP may also facilitate expression or function of the MC4R and that this could account for the marked obesity observed in a child with FGD type 2.^11^

When the clinical features of FGD types 1 and 2 were compared, striking distinctions were discovered in the age of presentation and height, but not in other aspects of endocrinology or body weight. The correlation between the estimated severity of the receptor defect *in vitro* and the degree of clinical severity remains poor. This was evident even for patients with the same MC2R mutation such as the most commonly occurring, S74I substitution in which the phenotype ranged from minimal to severe. Plotting height *vs.* age appears to be suggestive of a relationship between length of exposure to high ACTH/low glucocorticoid and tall stature, but we could not detect statistical significance.

Unusually tall stature has been described in many FGD type 1 cases in the literature,^1^,^3^,^4^,^28^ the cause of which is not known. *In vitro*, ACTH increases the development of a chondrogenic phenotype with an increase in proliferation and differentiation of chondrocyte precursors.^32^ Consequently, it is a reasonable hypothesis that ACTH at high concentrations could activate melanocortin receptors expressed in bone and the cartilaginous growth plate and stimulate growth.^33^ In adrenalectomized leptin-deficient mice given γ_2_-MSH, it was found that there were positive changes in linear growth parameters (both naso-anal and tibial length), suggesting that the melanocortin system plays a role in linear growth.^33^ Alternatively, glucocorticoid inhibits the synthesis of IGF binding protein 5 (IGFBP-5) in the osteoblast.^34^ Bone growth is stimulated by IGFBP-5, and thus conceivably cortisol deficiency results in a lack of negative inhibition and the consequent growth spurt seen in FGD type 1. No abnormality in the GH–IGF–I axis has been reported in FGD patients to date. It is interesting to note that MC2R knockout mice do not exhibit any significant difference in body length when compared with wild-type.^35^

Clinical observations suggest that replacement of glucocorticoid normalizes the advanced growth rate in FGD.^16^,^28^ In the reported cases, despite adequate hydrocortisone replacement, ACTH often remains elevated. The discordance of the plasma ACTH and bone growth suggest that exposure to cortisol could play a role in this. However, it is also very difficult to exclude the possibility that overtreatment with glucocorticoids is responsible for this deceleration in growth.

In conclusion, patients with familial glucocorticoid deficiency type 1 present later and have tall stature when compared with familial glucocorticoid deficiency type 2. This is consistent with the suggestion that prolonged ACTH excess or glucocorticoid deficiency increases linear growth. Familial glucocorticoid deficiency type 2, in contrast, presents earlier and appropriate treatment may be sufficient to prevent the enhanced growth rate seen in familial glucocorticoid deficiency type 1. There was no evidence that melanocortin 2 receptor accessory protein deficiency had any influence on any other physiological function beyond that seen with a defective melanocortin 2 receptor.
